# Orbital forcing of ice sheets during snowball Earth

**DOI:** 10.1038/s41467-021-24439-4

**Published:** 2021-07-07

**Authors:** Ross N. Mitchell, Thomas M. Gernon, Grant M. Cox, Adam R. Nordsvan, Uwe Kirscher, Chuang Xuan, Yebo Liu, Xu Liu, Xiaofang He

**Affiliations:** 1 State Key Laboratory of Lithospheric Evolution, Institute of Geology and Geophysics, Chinese Academy of Sciences,, Beijing, China; 2 Earth Dynamics Research Group, The Institute for Geoscience Research (TIGeR), Department of Earth and Planetary Sciences, Curtin University, Perth, WA Australia; 3 School of Ocean and Earth Science, University of Southampton, Southampton, UK; 4 Department of Earth Sciences, University of Hong Kong, Pokfulam, Hong Kong China; 5 Department of Geosciences, Eberhard Karls University Tübingen, Tübingen, Germany; 6 School of Geoscience and Survey Engineering, China University of Mining and Technology (Beijing), Beijing, China

**Keywords:** Palaeoclimate, Sedimentology

## Abstract

The snowball Earth hypothesis—that a runaway ice-albedo feedback can cause global glaciation—seeks to explain low-latitude glacial deposits, as well as geological anomalies including the re-emergence of banded iron formation and “cap” carbonates. One of the most significant challenges to snowball Earth has been sedimentological cyclicity that has been taken to imply more climate dynamics than expected when the ocean is completely covered in ice. However, recent climate models suggest that as atmospheric CO_2_ accumulates, the snowball climate system becomes sensitive to orbital forcing. Here we show the presence of nearly all Milankovitch (orbital) cycles preserved in stratified banded iron formation deposited during the Sturtian snowball Earth. These results provide evidence for orbitally forced cyclicity of global ice sheets that resulted in periodic oxidation of ferrous iron. Orbital glacial advance and retreat cycles provide a simple mechanism to reconcile both the sedimentary dynamics and the enigmatic survival of multicellular life during snowball Earth.

## Abstract (web summary)

Reconciling the Snowball Earth hypothesis with sedimentological cyclicity has been a persistent challenge. A new cyclostratigraphic climate record for a Cryogenian banded iron formation in Australia provides evidence for orbital forcing of ice sheet advance and retreat cycles during Snowball Earth.

## Introduction

Astronomical “Milankovitch” cycles^1^, related to changes in Earth’s orbit around the sun, exert a fundamental control on climate variability over tens to hundreds of thousands of years (kyr), with modulations in hundreds of kyr and even millions of years (Myr). Orbital forcing has presumably operated throughout Earth history with evidence found as old as ca. 2480 Myr ago^1–4^, and may have influenced the course of severe “snowball Earth” glaciations^5,6^, notably during the Cryogenian Period, about 720 to 635 Myr ago. Under snowball conditions, astronomical-induced variations in insolation due to Earth’s precession, obliquity, and eccentricity should be ongoing, but the range of associated climate variability and mechanisms in an ice-covered ocean are poorly understood. Recent ice sheet and atmospheric modeling results indicate that orbital forcing should be a viable climate driver under a wide range of atmospheric concentrations of CO_2_ between 0.1 and 200 mbar, and therefore was likely to have modulated ice sheet volume throughout much of the Cryogenian Period^5^.

While evidence for orbital forcing has been suggested in Cryogenian glacial successions^5^, evidence for multiple, internally consistent timescales of orbital forcing has not been demonstrated. This is likely because most glacial deposition occurs irregularly^7^, forming glacial diamictites (that is, lithified sediments comprising chaotic mixtures of a wide range of clast- and grain-size distribution) only rarely preserving stratification. One notable exception is the deglacial succession of the Elatina Formation with its tidal rhythmites^8^. Cyclostratigraphy—the study of orbital forcing in sedimentary successions—usually operates under the assumption of constant sediment accumulation (e.g., as expected in pelagic and hemi-pelagic facies^1^), so its application is hindered in sequences that do not preserve stratification (e.g., diamictite) or are characterized by frequent and abrupt changes in sediment accumulation rates (e.g., as in a delta front).

**Fig. 1:**
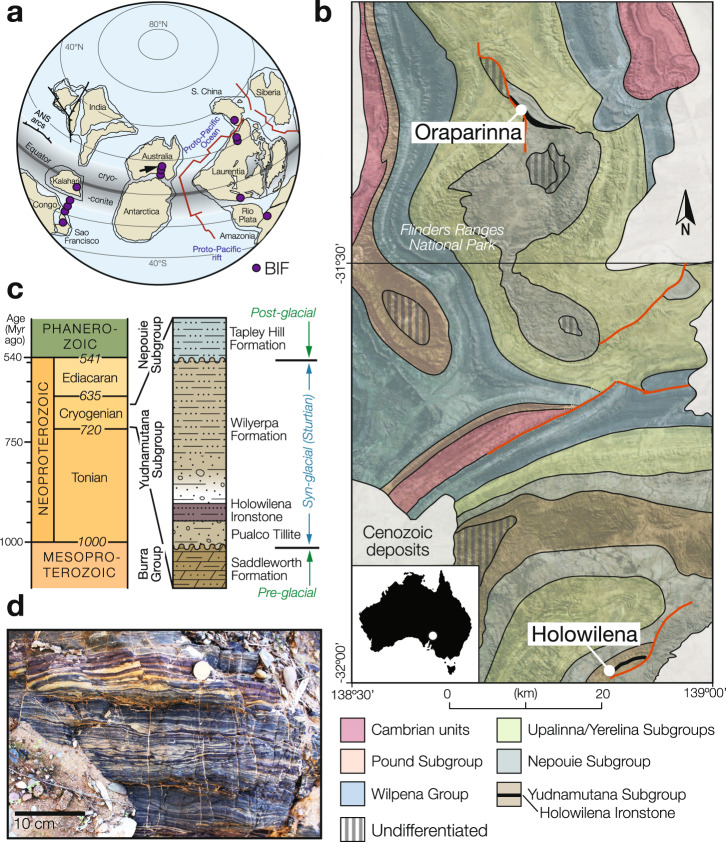
Spatial and temporal distribution of banded iron formation. **a** Palaeogeographic reconstruction ca. 700 Myr ago (updated from ref. ^44^) showing the locations of Cryogenian banded iron formation^9^ (BIF), and hypothesized cryoconite pans (gray) where cyanobacteria and eukaryotes may have taken refuge during snowball glaciation^12^. **b** Lithologic map of the two study locations at Oraparinna and Holowilena, South Australia (updated from ref. ^21^). **c** Simplified lithostratigraphy of the Sturtian glaciation in the Flinders Ranges, South Australia. **d** Fine-scale rhythmic banding observed in the Holowilena Ironstone at Holowilena.

Here we show that banded iron formation (BIF)^9,10^—well-stratified sedimentary rock containing abundant iron oxide—is ideally suited to cyclostratigraphic analysis because it is closely associated with glacial diamictites^9–12^, and deposited at a relatively constant rate when averaged over multiple depositional cycles as evidenced by the rhythmic banding^13,14^ on decimeter and centimeter (Fig. 1d; Supplementary Fig. 1c) and millimeter scales (Supplementary Fig. 2). Precambrian BIF has also been demonstrated to be both susceptible to orbital forcing and amenable to recording a clear orbital signal^4^. The occurrence of BIF in Neoproterozoic glacial deposits was initially conceived as a “last gasp” for global glaciation^15^ and thought to represent oxidation of hydrothermal iron accumulated within a snowball ocean after the re-emergence of open-ocean conditions^6,15^. However, this concept cannot readily explain how BIF in some cases is overlain or interbedded with glacial diamictites^11^. Most recently, BIF deposited during snowball Earth has been interpreted as the product of hydrothermal derived iron mixing with oxidized subglacial meltwater^16^.

## Results

### Stratigraphy and magnetic susceptibility

**Fig. 2:**
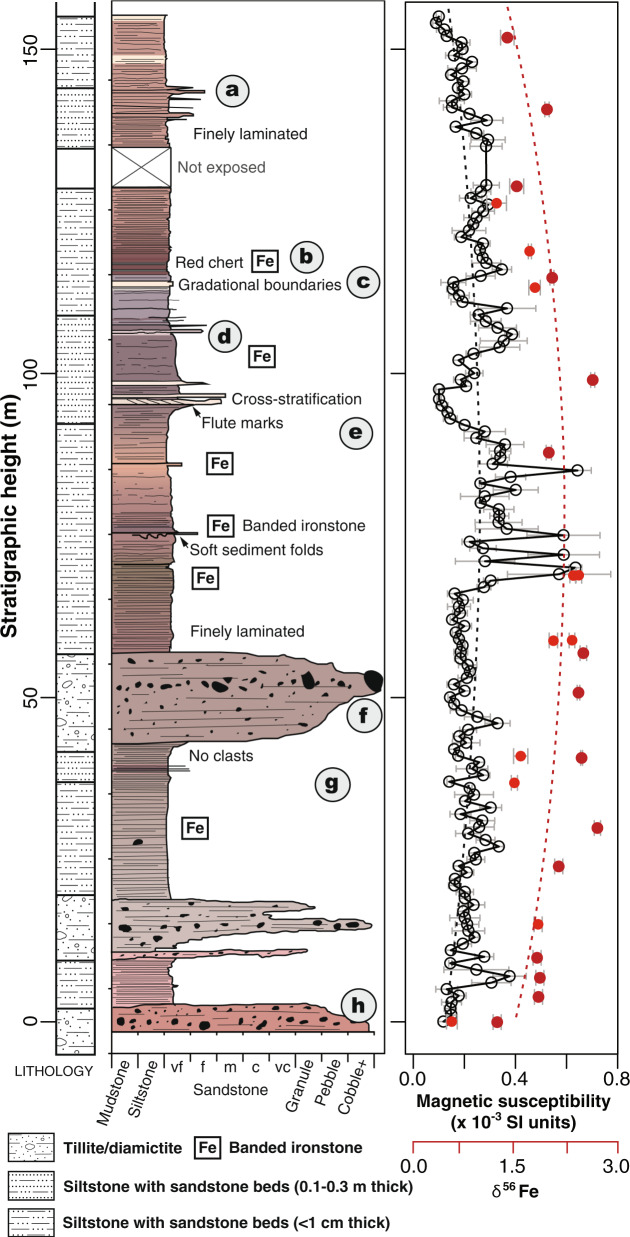
Stratigraphy, magnetic susceptibility, and δ^56^Fe isotope geochemistry at Oraparinna. (Left) Stratigraphic log featuring the key sedimentological facies of BIF. Lower-case letters refer to outcrop photographs (Supplementary Fig. 4). The colors shown, extracted from photographs, are accurate representations of the rock sequence variability. The lower faulted contact is at −12 m (see Supplementary Fig. 5 for additional field data). (Right) Magnetic susceptibility with 2σ uncertainty (black; Supplementary Table 1). δ^56^Fe isotope variability from two sections at Oraparinna: light red is the same section as ours^10^, and dark red is the section of ref. ^16^ ~6 km along strike (Supplementary Fig. 6); slightly less positive values at our section likely indicate relative proximity to the ice grounding line, consistent with more stratified diamictite than in the section of ref. ^16^. Best-fit degree-2 polynomials for each dataset (dashed lines). Note peak values for both trends occur in the middle of the succession. Rock magnetic experiments were performed on samples between 51 and 76 m (Fig. 5 and Supplementary Fig. 10).

We studied BIF sequences and their associated glacial deposits^11^ from the Sturtian glaciation in the Flinders Ranges of South Australia (Fig. 1; Supplementary Fig. 3). We performed time series analysis (Methods) that requires both adequate sampling resolution to detect high-frequency (short-wavelength) signals and long records for detecting low-frequency (long-wavelength) signals. To satisfy these requirements, we sampled Oraparinna Station (Figs. 1b, 2 and Supplementary Figs. 3–6; Supplementary Table 1) at 1-meter (m) intervals, allowing us to capture cycles with periods of several meters. A stratigraphic section at Holowilena, approximately 75 km south of Oraparinna (Fig. 1b; Supplementary Fig. 3), provided continuous exposure over a shorter interval and was sampled at 25 cm intervals to determine cycles with periods > 50 cm (Fig. 1b and Supplementary Figs. 3,7; Supplementary Table 2). Therefore, our sampling at Holowilena targeted high-frequency Milankovitch cycles (precession, obliquity, and short eccentricity) and at Oraparinna targeted low-frequency Milankovitch modulations (long eccentricity as well as ~1.2-Myr and ~2.4-Myr modulations of obliquity and eccentricity, respectively). This sampling strategy was designed around the outcrop conditions, where the river cut bank at Holowilena provides complete exposure over a short stratigraphic interval and the mountainside draw (or re-entrant) at Oraparinna provides incomplete but relatively consistent exposure over the entire BIF stratigraphy. The overall thicknesses and the lithofacies of the Holowilena BIF at both sections are strikingly comparable and nearly identical^10^. Given the proximity of Holowilena and Oraparinna and their near-matching stratigraphies, and that the eccentricity band spans target cycles at both localities, matching their short and long eccentricity are expected to yield comparable sediment accumulation rates. Faulting occurs ~1 km away from the Holowilena section^17^; nonetheless, we can confirm that the contact between the Holowilena BIF and the underlying Pualco Tillite is not faulted (Supplementary Fig. 1) and no significant faults were observed during detailed mapping of the ~18-m-thick section at Holowilena Creek, either in this study or in earlier investigations^10,11^.

Magnetic susceptibility—the measure of magnetizable minerals in a rock layer—was used as our cyclostratigraphic proxy for the BIF (“Methods”; Supplementary Fig. 8). Magnetic susceptibility is one of the more successful proxies used in astrochronology^18^, and our utilization is also motivated by our rock magnetic experiments, which indicate that the Holowilena BIF is highly variable in its hematite and magnetite content (Methods). Cyclostratigraphy has previously been applied to the Cryogenian interglacial (i.e., the ~10 Myr period between the Sturtian and Marinoan [~650–635 Myr ago] glaciations), yielding an astronomical interglacial chronology^2,19^. Cycles in magnetic susceptibility of Cryogenian BIF, if present, could be the result of either orbital forcing (i.e., allocyclic) or more autocyclic processes (e.g., glacial surges related to ice dynamics producing an equivalent sedimentary response). Therefore, it is critical to conduct rigorous time series analysis (“Methods”).

### Time series analysis

**Fig. 3:**
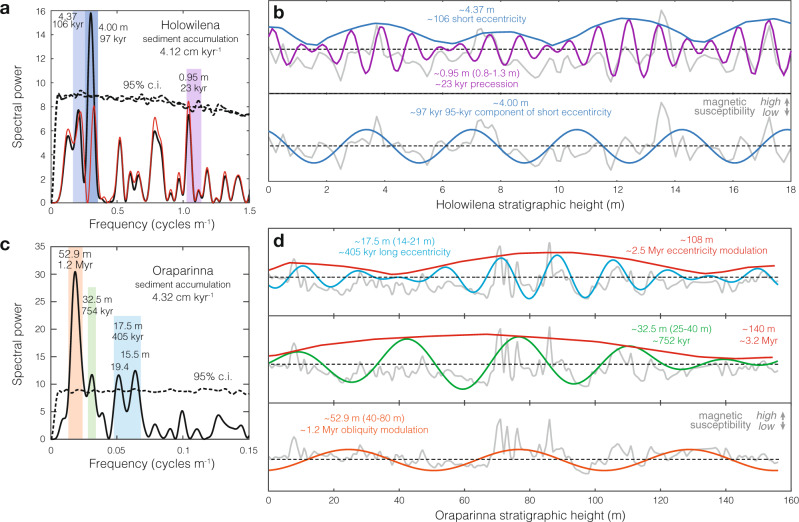
Time series analysis of iron formation. **a** Fast-Fourier transform (FFT) of magnetic susceptibility at Holowilena. Red spectra are FFT recomputed after subtracting the strongest signal (~4 m; ~97 kyr). Two distinct signals in the ~100 kyr bandwidth are shown as two overlapping transparent dark blue bands. **b** Bandpass filters of significant cycles identified with FFT at Holowilena. Raw magnetic susceptibility data are shown in gray. **c** FFT of magnetic susceptibility at Oraparinna. **d** Bandpass filters of significant cycles identified with FFT at Oraparinna. Envelopes of bandpass filters exhibiting amplitude modulation characterized using the Hilbert transform (Methods). Raw detrended data are shown as the gray line.

Our magnetic susceptibility data exhibit multiple statistically significant cycles at both sites (Fig. 3). At Holowilena, two cycles (periods of ~4 m and ~1 m) rise above the 95% confidence level (Fig. 3a, b). At Oraparinna, three cycles (periods of ~18 m, ~32 m, and ~53 m) rise above the 95% confidence level (Fig. 3c, d). The five bands identified (Fig. 3) can be compared with target astronomical cycles of Neoproterozoic age (Supplementary Fig. 9) by assuming a sediment accumulation rate (Supplementary Table 3). Beyond our measured section, the synglacial Sturtian stratigraphy at Oraparinna is complicated by local tectonics and salt diapirism^20^, so a regional sediment accumulation rate is inferred from the stratigraphic thicknesses at Holowilena.

Here, the synglacial Yudnamutana Subgroup, comprising the Pualco Tillite, Holowilena Ironstone, and Wilyerpa Formation (Fig. 1c), ranges in thickness along strike from 2068 to 2470 m (ref. ^21^). A high-precision zircon U-Pb age of 663.03 ± 0.11 Myr (2σ) was recently reported from a tuffaceous bed in this region (Copley, SA), roughly 80 m down sequence from the contact with the Wilyerpa Formation^22^, where lithofacies associations are comparable to those at Holowilena^11^. Assuming the accepted Sturtian onset age of 717 Myr^22^ and taking the well-constrained synglacial age of ~663 Myr for this area^22^, we assume a net sediment accumulation rate for the Yudnamutana Subgroup of 3.7 to 4.4 cm kyr^−1^, a firm starting point for matching observed cycles to putative matches with astronomical cycles. Using this range as an initial guide, we selected the rate for each stratigraphic section that minimizes the net misfit between all observed cycles and their respective target cycles. We found that sediment accumulation rates inferred for both BIF sections, when matching observed cycles to astronomical targets (~4.2 cm kyr^−1^; Supplementary Table 3), are self-consistent and within the plausible range of sediment accumulation rates for the Yudnamutana Subgroup. Intervals of stratified diamictite at Oraparinna (Fig. 2) appear to exhibit similar relatively constant sediment accumulation rates (Fig. 3d).

At Holowilena, the shortest cycle identified (~1 m) yields an inferred ~23 kyr period consistent with orbital precession, the highest-frequency astronomical cycle. The longest cycles detected at Holowilena are in the ~100 kyr band: (i) amplitude modulation of the presumed precession signal yields an envelope reminiscent of short ~95–125 kyr eccentricity (see below), and (ii) a narrow and unmodulated cycle at ~97 kyr that most likely represents the strong 95-kyr component of the 4-component short eccentricity signal. At Oraparinna, the shortest cycle represents ~405 kyr long eccentricity, Earth’s “metronome”^23^, considered the most stable astronomical period through time^1,24^. Thus, the short eccentricity signal is identified in the high-resolution section and long eccentricity is identified in the longer section, providing an overlap of the results in the eccentricity bandwidth. The longest, high-confidence cycle identified at Oraparinna (53 m) translates to a period of ~1.2 Myr, close to that of the long-term, modulation of obliquity^1^ or the ~1 Myr modulation of precession^25^. Additionally, amplitude modulation of the presumed long eccentricity signal of Oraparinna yields an envelope with a period of ~2.5 Myr, close to the ~2.4 Myr modulation of eccentricity^1^. The ratios of the cycles identified at the two sections of Holowilena BIF are thus internally consistent with each other when interpreted as the hierarchy of all known Milankovitch cycles and modulations (Fig. 3). The exclusive absence of a high-frequency obliquity signal at ~30 kyr could be explained by Australia’s low palaeolatitude during deposition of the Holowilena BIF (Fig. 1a).

**Fig. 4:**
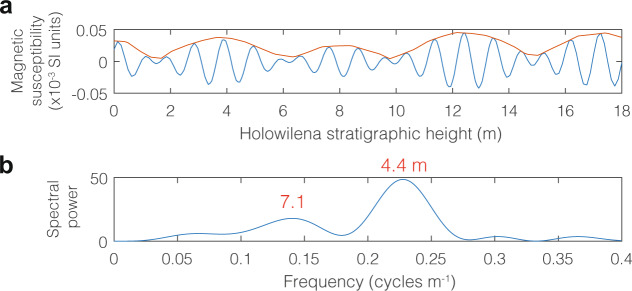
Hilbert transform of putative precession signal in order to extract putative eccentricity signal. **a** Hilbert transform (red) extracted from precession bandpass filter (blue; Fig. 3b). **b** Fast-Fourier transform (FFT) of the Hilbert transform, indicating power in the eccentricity band (compare to Fig. 3a), as expected.

The magnetic susceptibility data from Holowilena pass one of the most diagnostic tests for orbital forcing: eccentricity extracted from precession. Due to its effect on Earth’s equinoxes, eccentricity primarily affects insolation due to its amplitude modulation of precession^1,26^. Thus, in Phanerozoic cyclostratigraphy, the eccentricity signal is usually extracted from that of precession^1^. Using the Hilbert transform for the extraction (“Methods”), the envelope of the putative precession signal (~23 kyr) is systematically amplitude-modulated in bundles of ~5 cycles, consistent with precession modulated by eccentricity (Fig. 3b). Spectral analysis of the ~23 kyr Hilbert transform yields power in the short eccentricity band (Fig. 4), confirming that an eccentricity signal can be extracted from precession.

It should be noted that with ~5 precession cycles per short eccentricity cycle (Figs. 3b, 4), our results for the middle of the Sturtian glaciation imply anomalously slow precession compared to astronomical models^27–29^ that estimate ~6.5 precession cycles per short eccentricity cycle at this age. There are several possible explanations to consider. Firstly, it is possible that available astronomical models^27,28^, although generally in agreement with each other, are inaccurate by this age. However, cyclostratigraphic data as old as 1.4 Gyr ago appear to support the long-term astronomical model trajectories^29^. Secondly, it is possible that missing precession cycles per short eccentricity cycle in minor unconformities or cyclic changes in sedimentation rate related to precession amplitude may distort the observed ratio with eccentricity. However, there are no unconformities in either of the studied sections and the strong evidence of eccentricity-modulated precession suggests that we have resolved the relative ratio of eccentricity and precession at this age. Ruling out problems with either the models or the data, we offer a third, albeit more speculative, option. It is feasible that this anomaly is related to the conversion of large volumes of seawater into extremely thick sea ice and continental ice sheets during the Cryogenian. Such a transfer of mass away from Earth’s spin axis would reduce the moment of inertia and, in order to conserve angular momentum, have temporarily slowed Earth’s rate of rotation. As precession is related to rotation rate but eccentricity is not, this mechanism might explain the unexpected bundling observed by us for this age. Then, upon snowball deglaciation, Earth’s rotation would have resumed its previously fast rate of rotation. Importantly, such an effect on rotation has been modeled for Pleistocene glaciations^30^ and would presumably have been much more substantial on snowball Earth. Garnering corroborating evidence and testing such a possibility with modeling is beyond the scope of this study and warrants further investigation.

In summary, we find that the origin of magnetic susceptibility cycles in the Holowilena Ironstone (Fig. 3) are best explained by orbital forcing based on (i) the close matches with all Milankovitch cycles and modulations when assuming sediment accumulation rates constrained by independent geochronology; (ii) the reasonable and consistent implied sediment accumulation rates; and (iii) the striking evidence for eccentricity-modulated precession. If cycles in magnetic susceptibility in the Holowilena BIF were not orbital but autocyclic in origin, all observations would have to be regarded as coincidental.

## Discussion

**Fig. 5:**
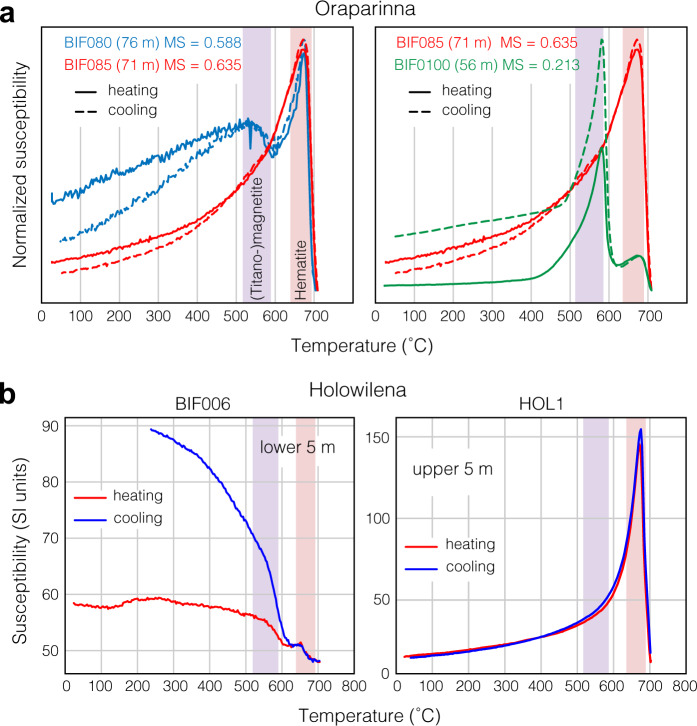
Thermal susceptibility experiments. **a** Oraparinna. Thermal susceptibility comparison between samples across a short eccentricity cycle (~100 kyr; left) and a long eccentricity cycle (~400 kyr; right). Stratigraphic heights relate to the Oraparinna section (Fig. 2). Unblocking temperatures near ~675 ˚C and ~585 ˚C indicates the presence of hematite and magnetite, respectively. Note hematite is dominant, but magnetite content is variable. Hematite-pure lithologies yield high magnetic susceptibilities and hematite-poor lithologies yield low susceptibilities. **b** Holowilena. Sample BIF006 from the lower 5 m of the section (Supplementary Fig. 1) exhibits a dominant magnetite peak and subordinate hematite peak, whereas sample HOL-1 from the upper 5 m contains pure hematite. Supplementary Fig. 10 shows the results of other, corroborating rock magnetic experiments. Purple and red vertical bands in the background show typical ranges of (titano-)magnetite and hematite, respectively.

Assuming orbital forcing controls variability in magnetic susceptibility in the BIF, the question then becomes by what mechanism the signal is recorded. Rock magnetic experiments of our ironstone samples (“Methods”) demonstrate that magnetic susceptibility varies due to Fe-oxide mineralogy, specifically, the relative proportions of hematite and magnetite. For a sample that yields one of the highest susceptibility values at Oraparinna (~71 m; Fig. 2), thermal magnetic experiments suggest that the magnetic phase comprises almost purely hematite with minor magnetite (Fig. 5; Supplementary Fig. 10). This result is surprising as the susceptibility of magnetite is two orders of magnitudes stronger than that of hematite, implying a dominance of hematite in the Holowilena Ironstone. Fe-oxide mineralogy varies more over the ~15 m stratigraphic interval than it does over the ~5 m interval (Figs. 2, 5a), consistent with variability in susceptibility driven predominantly by long (405 kyr) and short (~100 kyr) eccentricity cycles, respectively (Fig. 3). Samples collected at the base and the top of the Holowilena section that exhibits an upsection increase in susceptibility (Supplementary Fig. 8) yield magnetite- and hematite-dominated thermal susceptibility signatures, respectively (Fig. 5b). This confirms that susceptibility in the Holowilena BIF is dominantly controlled by pure (high susceptibility) or subordinate (low susceptibility) proportions of hematite, just as we observed at Oraparinna (Fig. 5a). Samples in which magnetite dominates, consistent with petrographic observations (Supplementary Fig. 2), exhibit irreversible thermal susceptibility experiments (BIF0100 and BIF006 in Fig. 5), suggesting the presence of clays that convert to magnetite upon cooling^31^; as clays are paramagnetic and would contribute little to susceptibility compared with ferromagnetic minerals, their presence in such samples is consistent with the observation that hematite-poor and magnetite-rich lithologies have low susceptibility.

**Fig. 6:**
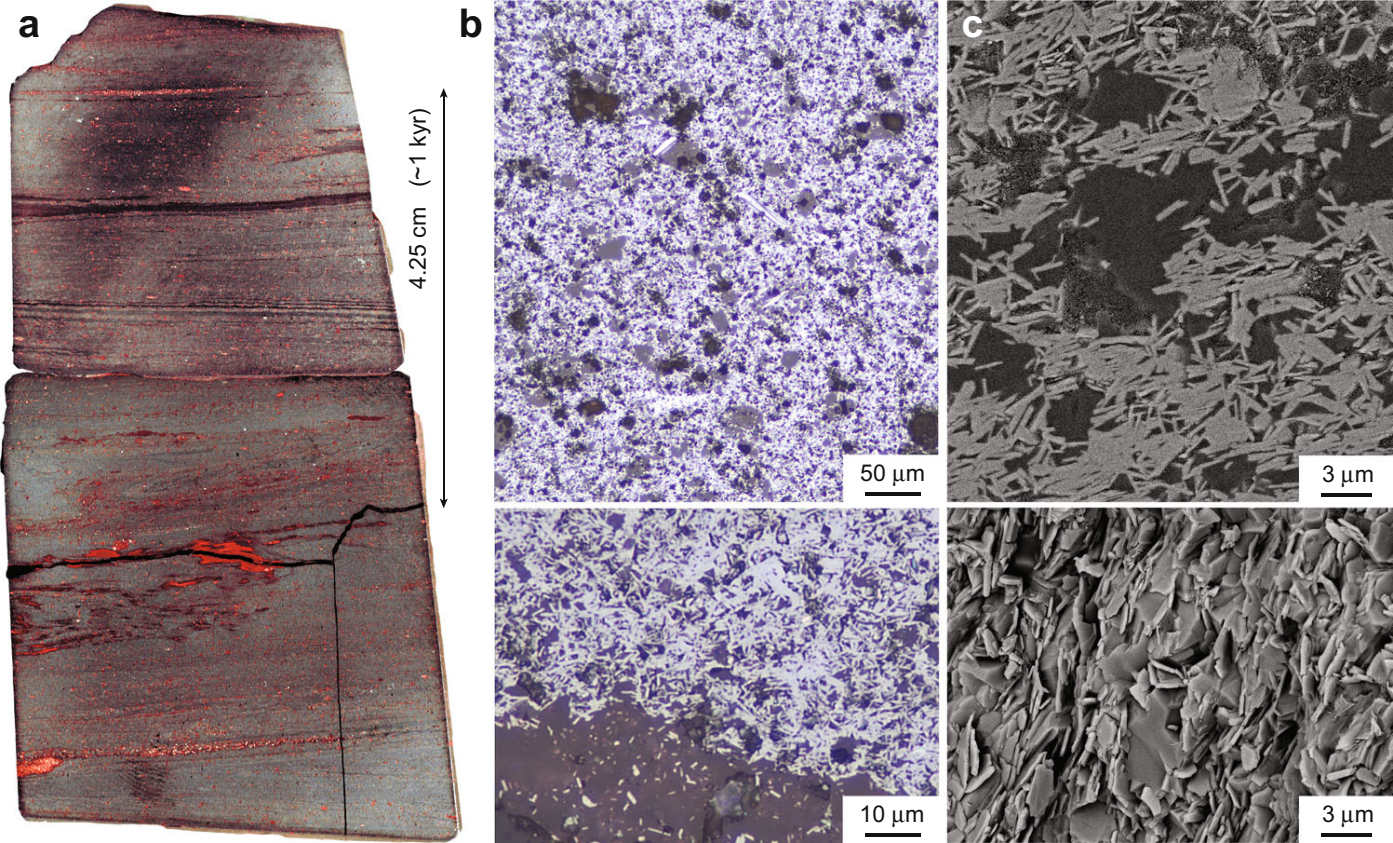
Petrography of iron formation. **a** High susceptibility, hematite-pure hand sample (HOL-1) from Holowilena (Fig. 5b). Note red jasper is laterally discontinuous and interpreted to be chemically precipitated. **b** Photomicrographs under reflected light show randomly oriented euhedral hematite laths (white) in a quartz, chert, and clay matrix (gray). Note large gray intraclast at the bottom, also containing hematite laths. **c** SEM images of polished thin section (top) and rock chip (bottom) exhibiting randomly oriented hematite laths in two and three dimensions, respectively. The textures strongly suggest that the hematite is authigenic (i.e., chemically precipitated) and not detrital.

Hematite in the Holowilena BIF is interpreted as the product of the oxidation of dissolved Fe^2+^ due to free O_2_ (ref. ^13^). Detailed petrography of a high susceptibility, hematite-pure sample from Holowilena (HOL-1) reveals pore-filling euhedral hematite laths that suggest the hematite is authigenic and precipitated out of the snowball ocean due to oxidation (Fig. 6). Two possible sources of oxygen must be considered. According to a “hard” snowball scenario where temperatures are so low that surface melting is absent and ablation is controlled by sublimation^12^, a viable mechanism for orbitally paced oxygenation of a snowball ocean would be meltwater injection when an ice sheet margin reached tidewater during times of ice advance. In this case, meltwater discharge occurs when the ice margin advances to the coast, but not when it retreats inland^32^. Alternatively, according to a “soft” or more dynamic snowball scenario, air-sea gas exchange would allow for the oxidation of the dissolved Fe^2+^ pool during periods of glacial retreat if ice-free regions of sea ice (i.e., polynyas) open up^11^. These two models thus make testable, opposite predictions, with oxygenation of the snowball ocean occurring during glacial advance (tidewater model) and retreat (polynya model).

Depending on whether the hematite>magnetite (high magnetic susceptibility) phase of each orbital cycle correlates with glacial advance or retreat can potentially test between the tidewater and polynya models and their respective oxidizing agents (glacial meltwater and the atmosphere, respectively). In addition to Fe-oxide mineralogy, magnetic susceptibility also varies consistently with δ^56^Fe isotope variations^10^ (Fig. 2), which are redox-sensitive and have been interpreted in terms of glacioeustasy^10^ and subglacial meltwater oxygenation^16^. The increase in δ^56^Fe values in the lower-to-middle Holowilena BIF corresponds with a sedimentological change from ice-proximal (diamictite/dropstone-dominated) to ice-distal (mudstone with rare dropstones) facies (Fig. 2). This trend towards such uniquely positive δ^56^Fe values has been interpreted as due to either a decrease in oxidized meltwater discharge causing a return to anoxia^16^ or the shoaling of the Fe chemocline^10^; both interpretations are consistent with glacial-retreat/sea-level-rise and the observed facies changes. This increasing isotopic trend corresponds with increasing susceptibility, which is attributable to a rising abundance of hematite as constrained by rock magnetic experiments (Fig. 5) and petrography (Fig. 6). As hematite appears to increase during periods of glacial retreat and not glacial advance, this may preliminarily indicate that the O_2_ is dominantly supplied from polynyas during glacial minima and not meltwater discharges into tidewater during glacial maxima. It should be noted, however, that assigning glacial phase relationships to orbitally forced lithologic variations is difficult even for Phanerozoic climate records^26^ and since both oxygenation models are viable, further detailed and interdisciplinary work is required to test between their contrasting climatic predictions. Either way, this strong redox gradient observed in Cryogenian BIF requires both glacial advance and retreat to explain the cycles. Evidence for such dynamic, significant, and sustained eustasy and/or redox variability carries several important implications for snowball Earth climatology.

The observed orbital forcing reported here during the Sturtian glaciation helps resolve two fundamental enigmas about snowball Earth. Firstly, widespread sedimentological variations within Cryogenian diamictites were difficult to reconcile without a hydrological cycle. These features have been interpreted by some as evidence against a “hard” snowball, inspiring alternative models that involved partially open-ocean conditions^33–36^. Documenting orbital cycles corroborates models that suggest snowball Earth ice sheets were sensitive to orbital forcing^5^ and accounts for well-documented Cryogenian sedimentological variations and cycles^5,37^ with or without partially open-ocean conditions. Secondly, molecular phylogenetic clocks estimate a Sturtian origin for several crown-group metazoans^38^, prompting the question of how life survived snowball Earth^6,39^. Multiple refugia have been proposed^12^, but the operation of orbital forcing provides previously unrecognized and globally available refugia in which diversifying eukaryotes could endure severe, protracted, and repeated snowball glaciations. We conclude that BIF deposition during snowball glaciation and its associated episodic oxygenation of marine environments were controlled by orbitally induced advance and retreat of global ice sheets.

## Methods

### Magnetic susceptibility

Magnetic susceptibility was measured on surface exposures of the Holowilena Ironstone using a standard-calibrated KT-10 field susceptibility meter. Cycles extracted from time series generated with such a field meter and lab-based measurements via a Kappabridge instrument have been shown to be consistent with each other^40^. Measurements were made on unique facets of a single stratigraphic horizon. Typically, ≥4 measurements were averaged at each stratigraphic level, above which precision became saturated. Field-based measurements of magnetic susceptibility allowed multiple measurements to be acquired along discrete stratigraphic layers, thereby both reducing analytical uncertainty and allowing the natural variance to be constrained. In preparation for time series analysis, long-term trends in magnetic susceptibility for each section were removed including a linear regression for Holowilena and a degree-2 polynomial for Oraparinna (Supplementary Fig. 8).

### Grayscale image analysis of thin sections

Two large format (5 cm) thin sections from Holowilena were made and imaged under plane-polarized light (Supplementary Figs. 2 and 11). Photomicrographs were stitched together using the GigaPan^©^ Software. Grayscale data were generated using the ‘Plot Profile’ function of the freely available ImageJ software (Supplementary Fig. 11). Pixel values were converted into stratigraphic thickness (mm) according to the length of the record selected along the thin section. In preparation for time series analysis, BIF1 (top in Supplementary Fig. 11) was converted to log-scale and did not require detrending, and BIF3 (bottom in Supplementary Fig. 11) was detrended with a degree-3 polynomial fit, which likely represents a millennial-scale cycle.

### Time series analysis

We conducted time series analysis using the Fast-Fourier transform (FFT)^41^ to test for the presence of any significant magnetic susceptibility cycles. Spectral analyses were conducted in the stratigraphic domain, which yielded a set of spectral peaks representing cycles per stratigraphic meter. For the FFT, the spectral power used is the complex conjugate of the Fourier coefficients, normalized to unit mean power^41^. We evaluated the significance of the FFT spectral peaks using a Monte Carlo routine to simulate noise^41^. FFTs were performed on each of these 1000 randomly generated time series; a 95% confidence level was approximated for each frequency by calculating three times the mean power^41^. We interpret spectral peaks rising above this 95% confidence level as statistically significant, a commonly used statistical threshold for identifying non-random occurrences. Based on the FFT results, we ran bandpass filters with Gaussian windows to encapsulate the significant peaks identified. Amplitude modulation of a bandpass, if present, was analyzed using the approach of Grippo et al.^42^ in which a Hilbert transform is used to find the enveloping curve of a bandpass signal (in the time domain); an FFT was performed on the enveloping curve to identify the cycles of amplitude modulation of the shorter-wavelength signal. Since eccentricity is rectified as climate change by its amplitude modulation of precession^1^, the Hilbert transform can test for evidence of such diagnostic spectral behavior.

Observed cycles in the stratigraphic domain were converted into the time domain by assuming a sediment accumulation rate considered reasonable for Cryogenian strata in the Flinders Ranges (see main text). Resulting observed cycle periods were then compared to target cycle periods based on models of solar system evolution. Due to decelerating rotation and increasing Earth-moon distance, respectively, both precession and obliquity are expected to systematically lengthen over geologic time^27^ (Supplementary Fig. 9; Supplementary Table 3). On the other hand, the periods for short (95 and 124 kyr) and long (405 kyr) eccentricity are considered constant “metronomes”^1,23^. After using the inferred range of feasible sediment accumulation rates of 3.7 to 4.4 cm kyr^−1^ as an initial guideline (see main text), we selected the sediment accumulation rate for each stratigraphic section that yielded the minimum net misfit^43^ between all observed cycles and their respective target cycles (Supplementary Table 3).

### Rock magnetic experiments

Selected samples from Oraparinna and Holowilena were ground to a fine powder. Magnetic susceptibility of the samples was monitored on heating from room temperature to 700 °C and subsequent cooling to room temperature (at heating and cooling rates of ~11 °C min^−1^) using an AGICO KLY-4S Kappabridge Susceptibility instrument. A portion of these samples were also used for room temperature isothermal remanent magnetization (IRM) acquisition and hysteresis loop experiments on a Princeton Measurements Corp. Model 3900 Vibrating Sample Magnetometer (VSM). IRM of the samples was acquired and measured at sixty field steps on a logarithmic scale ranging from 1 mT to 1 T. Hysteresis loops of the samples were measured at 5-mT field steps, with the applied field ranging between −1 T and +1 T. Thermal susceptibility results are shown in Fig. 5. All other rock magnetic experiments are summarized in Supplementary Fig. 10. Oraparinna rock magnetic samples come from BIF080, 76 m level (Figs. 2, 5a); BIF085, 71 m level (Figs. 2, 5a); BIF100, 56 m level (Figs. 2, 5a). Holowilena rock magnetic samples come from (−31.987226°N, 138.849969°E): BIF006, lower 5 m of BIF in Holowilena logged section (Supplementary Figs. 1, 8; Fig. 5b); HOL-1, upper 5 m of BIF in Holowilena logged section (Fig. 5b; Supplementary Fig. 8).

## Supplementary information

Supplementary Information

## Data Availability

All magnetic susceptibility data are provided in Supplementary Tables 1 and 2.

## Code availability

Matlab scripts used for time series analysis are available from the authors upon reasonable request.
