# Supplementary material for: Orbital forcing of ice sheets during snowball Earth
Source: Nat Commun. 2021 Jul 7;12:4187. doi: 10.1038/s41467-021-24439-4 (PMC8263735; doi:10.1038/s41467-021-24439-4)
Supplement: Supplementary file 1 — Supplementary Information [file 41467_2021_24439_MOESM1_ESM.pdf]

Supplementary Information for

Orbital forcing of ice sheets during snowball Earth

R.N. Mitchell*, T. M. Gernon, G.M. Cox, A.R. Nordsvan, U. Kirscher, C. Xuan, Y. Liu, X. Liu, and X. He

This PDF includes:

Supplementary Figs. 1 to 11

Supplementary Tables 1 to 3

Supplementary references

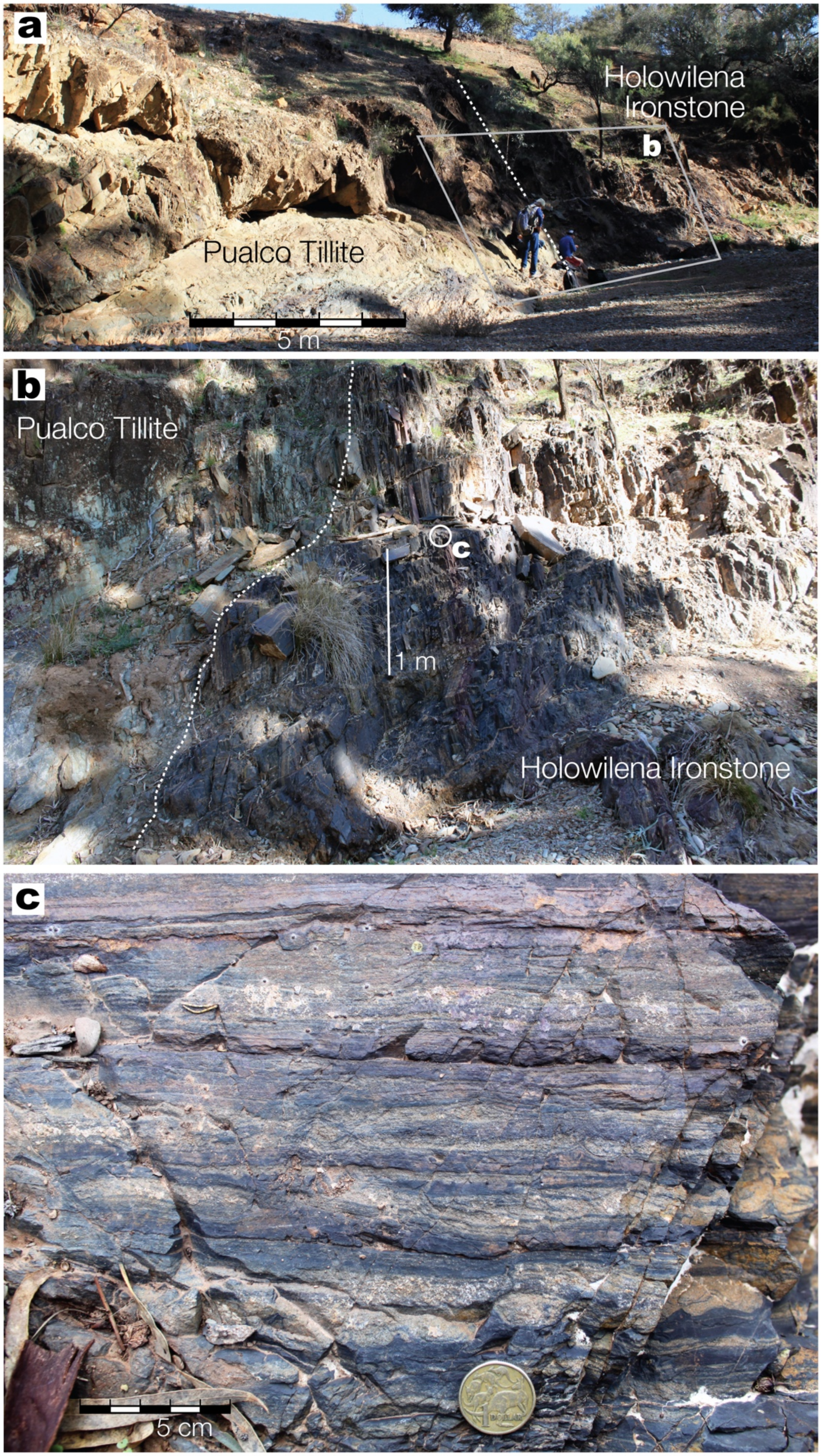

Supplementary Fig. 1. Field photographs showing details of the studied section at Holowilena. a Contact between the Holowilena Ironstone and the underlying Pualco Tillite, indicating inset of photo in **b** is not faulted. **b** Close-up of non-faulted contact shown in **a**, indicating inset of the photo in **c**. **c** BIF defined by regular thin beds (coin is 2.5 centimetres in diameter).

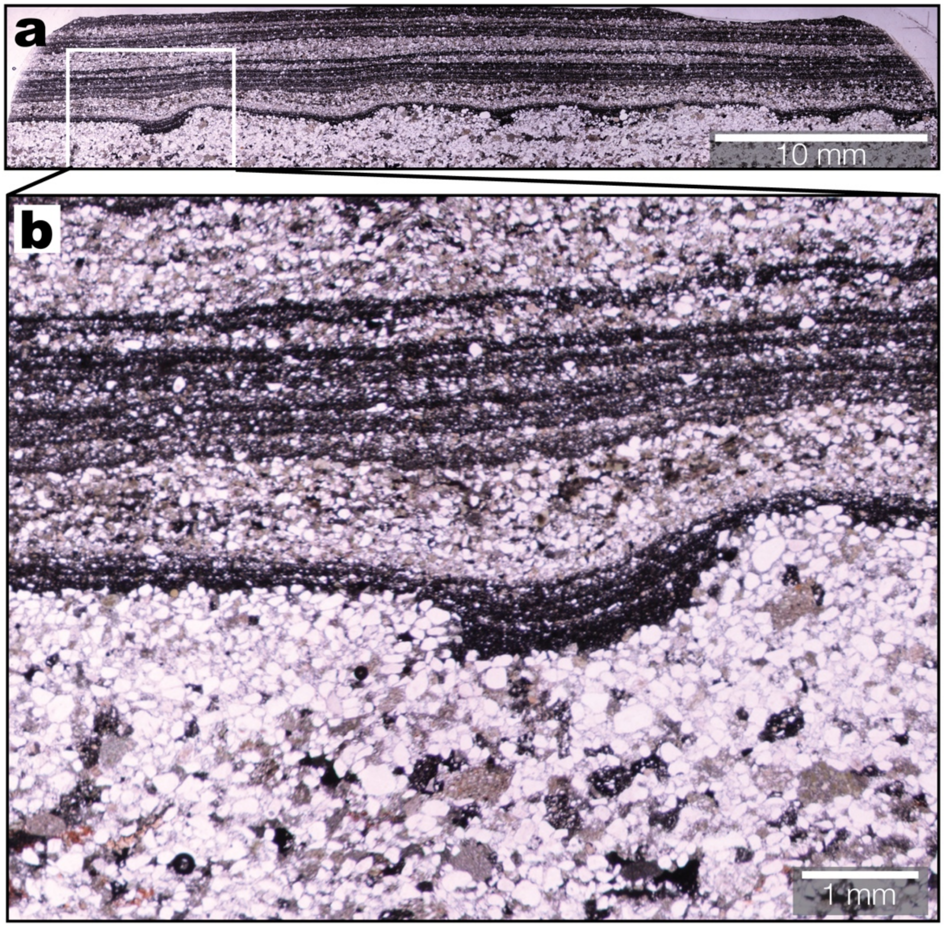

Supplementary Fig. 2. Petrography of the lower Holowilena Ironstone at Holowilena. Portion of a photomicrograph constructed by stitching together 20 discrete photomicrographs acquired under plane polarized light. Laminated BIF (sample BIF006) from the lower 5 metres of the Holowilena section (Supplementary Fig. 1b), which is characterized by relatively low magnetic susceptibilities compared to the upsection increase (Supplementary Fig. 8). Fe-oxides are opaque (dark) and clastic grains (quartz, clays, etc.) are light. Petrography suggests that the Fe-oxide mineralogy in this sample is dominantly magnetite on the basis of the following observations: (i) the mineral is opaque, unlike hematite, which is more commonly translucent; (ii) the mineral is black under plane polarized light, unlike hematite which is commonly red to brown; (iii) the mineral does not exhibit pleochroism unlike hematite, which is anisotropic. The combination of these three observations, coupled with thermal susceptibility measurements (Fig. 5b), strongly indicates the lower Holowilena Ironstone is dominated by magnetite. **b**, Laminated BIF overlies fine-medium grained sandstones with an erosive contact and shows that the Fe-mineral phase infills small-scale scour structures, with the Fe-oxide apparently thickening into the depression. The finely laminated nature of the magnetite-rich phase forms very well defined and regular alternations with siltstone on a ~200 μm scale. Supplementary Figure 11 displays the lithologic cycles across the entire thin section.

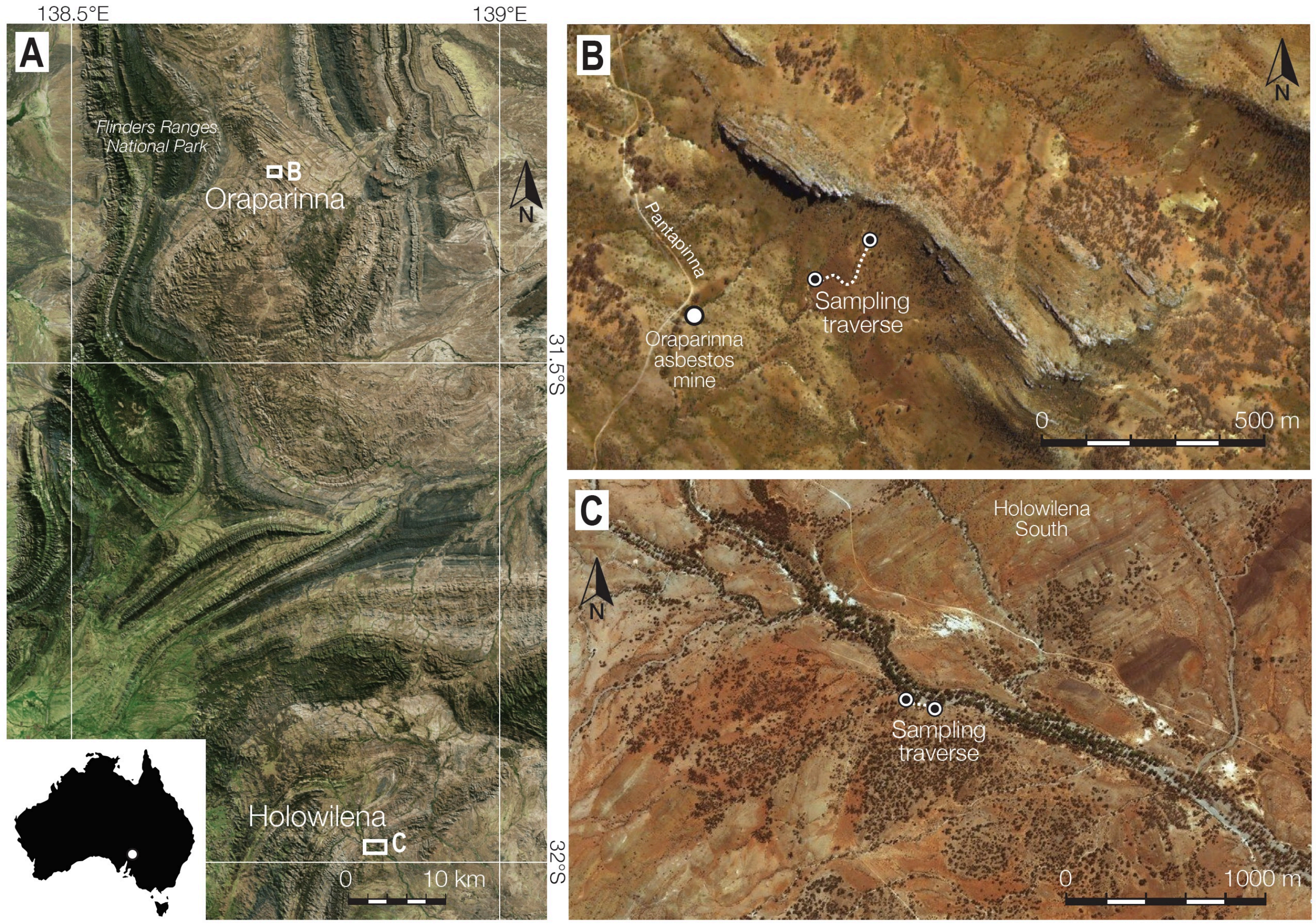

Supplementary Fig. 3. Sampling localities in the Flinders Ranges of South Australia. **a** Overview map showing the locations of both studied sections. **b** Map of the studied section at Oraparinna. **c** Map of the studied section at Holowilena.

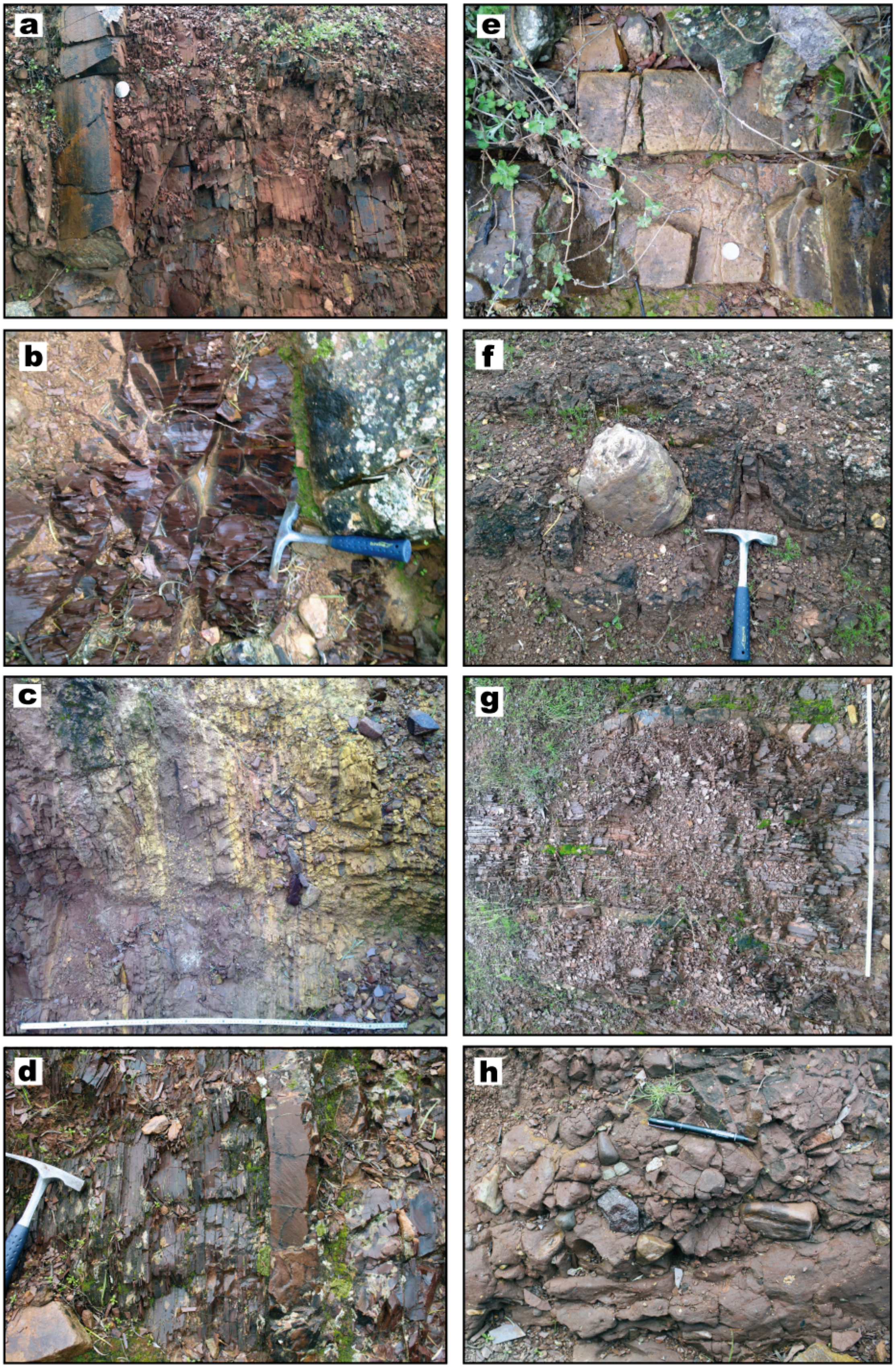
a
e
b
f
c
g
d
h

(continued from page above)

Supplementary Fig. 4. Field photographs showing details of the studied section at Oraparinna. **a** Interbedded sandstone and BIF. **b** BIF locally rich in chert showing a well-developed conchoidal fracture pattern. **c** Alternating red and yellow bands define the upper part of the BIF sequence at Oraparinna (note scale bar is 1 metre). **d** Medium bedded sandstones with well-developed scour marks and flute casts at the base. **e** Close-up of a sandstone bed containing cross stratification. **f** Striated boulder in the mixed diamictite-BIF unit near the base of the sequence (see Fig. 2). **g** BIF defined by regular thin beds (again, scale bar is 1 metre). **h** Diamictite containing rounded clasts (pen is 14 cm long). See Figure 2 in the main paper for the stratigraphic position of each image.

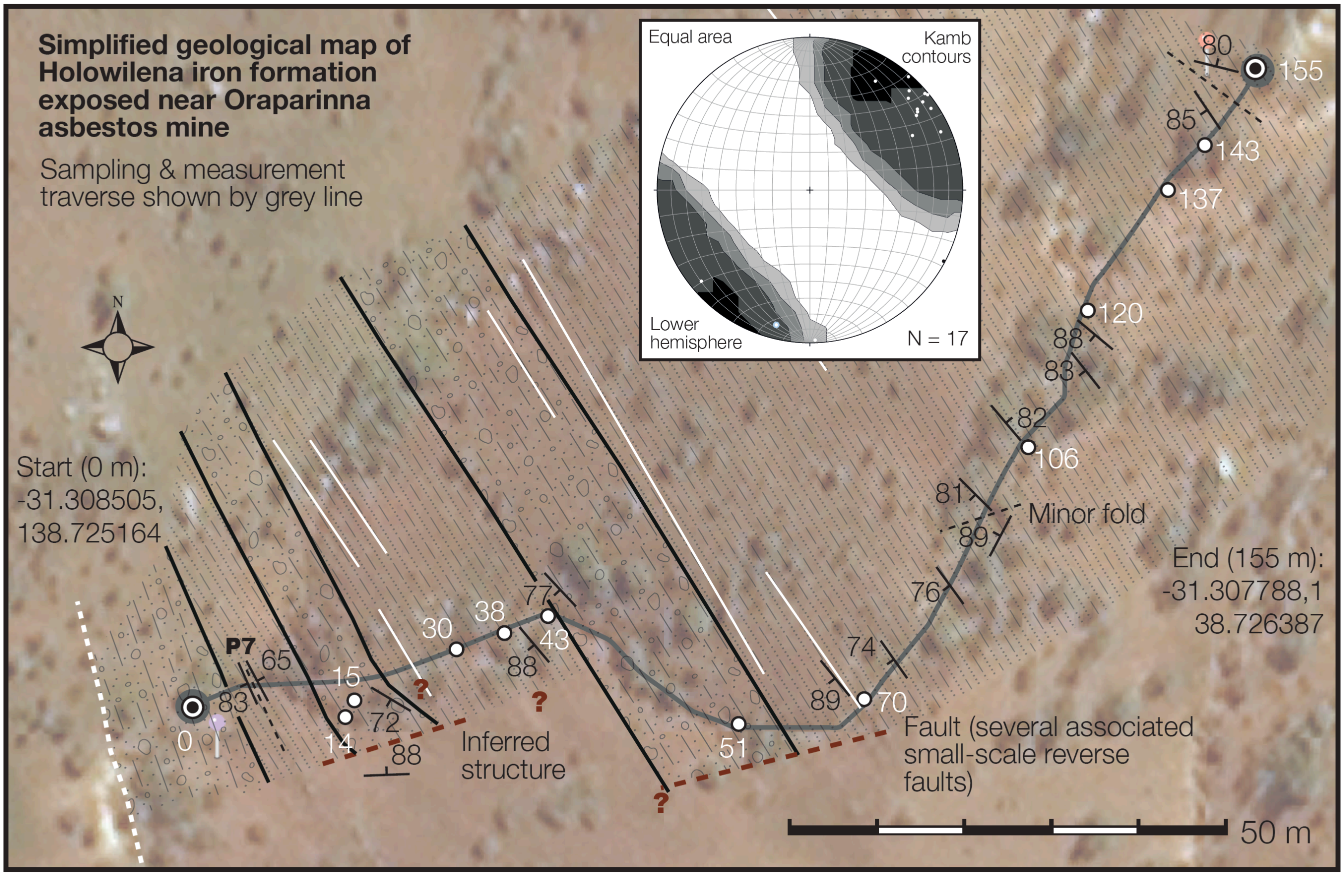

Supplementary Fig. 5. Simplified geological map of the Holowilena iron formation exposed near Oraparinna asbestos mine. Sampling and measurement traverse along the stream section is shown by the gray line. The accompanying stereonet shows the poles to bedding orientations as white circles (n = 17), with Kamb contours showing point density.

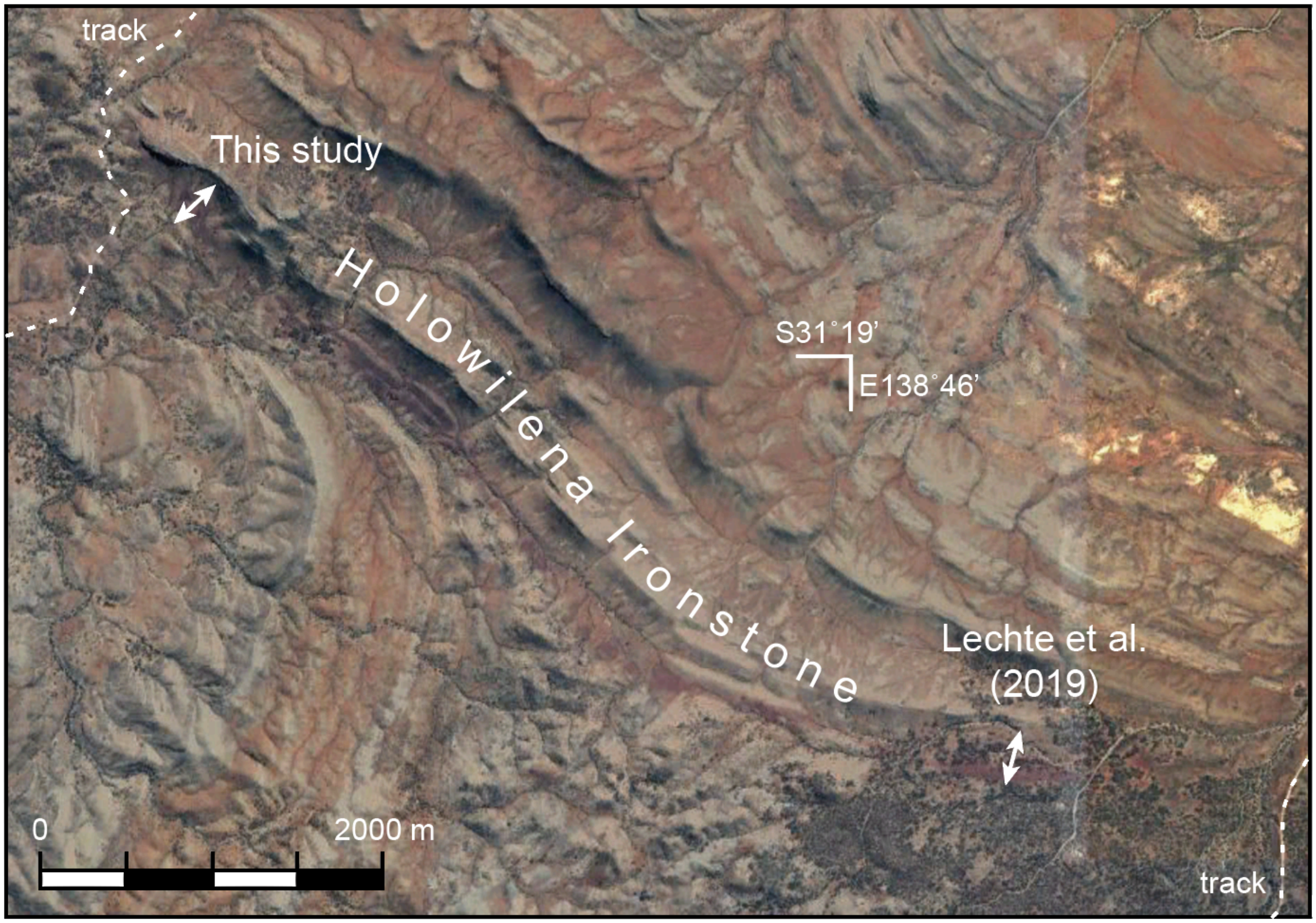

Supplementary Fig. 6. Sections with δ^{56}Fe data at Oraparinna. Stratigraphic sections of the Holowilena Ironstone at Oraparinna along strike from each other ~6 km apart: that is this study and Cox et al.[1] in the west, and that of Lechte et al.[2], in the east. All associated δ^{56}Fe data is depicted in Figure 2 of the main paper.

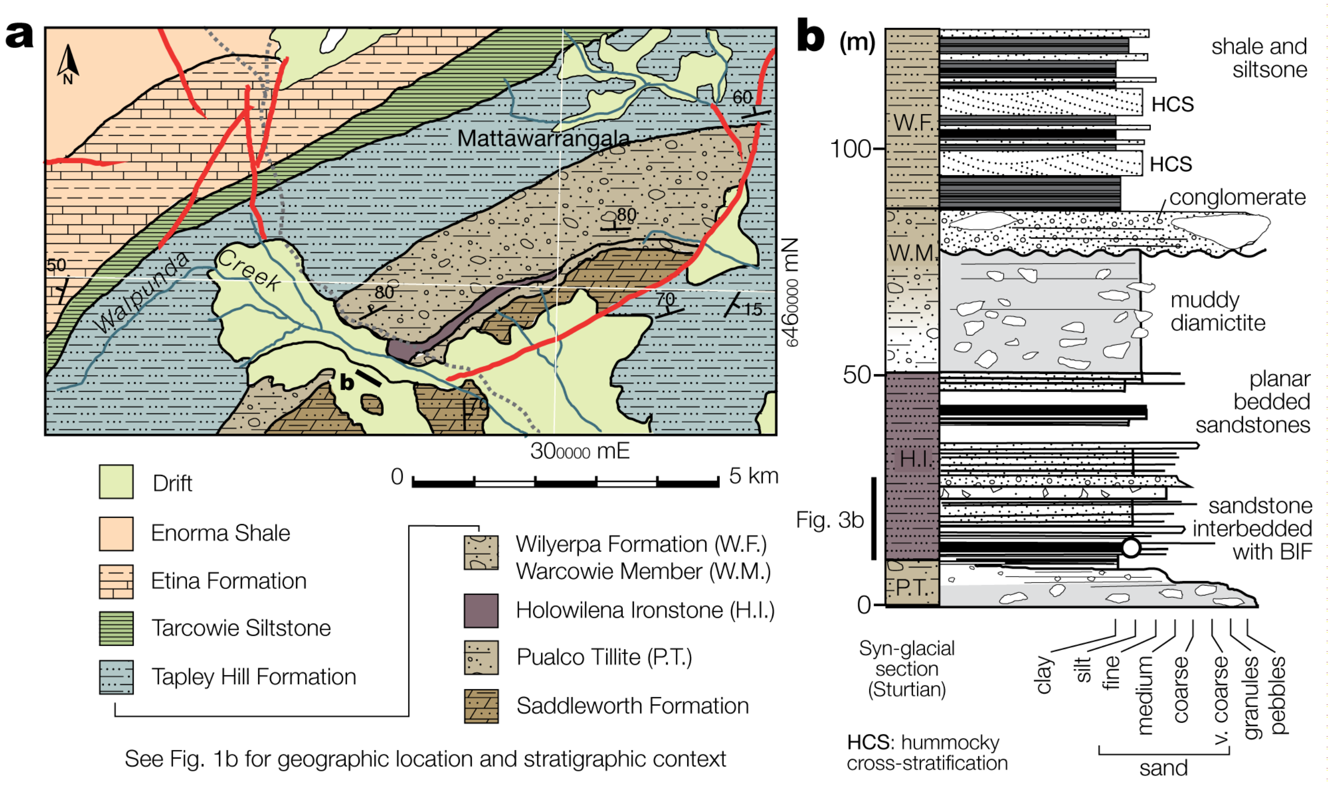

Supplementary Fig. 7. Geologic and stratigraphic context of the Holowilena section. a Geologic map of the Holowilena section (see also Figure 1b) updated from ref. [3]. **b** Stratigraphic context of the Holowilena section (shown in Figure 3b) updated from ref. [4].

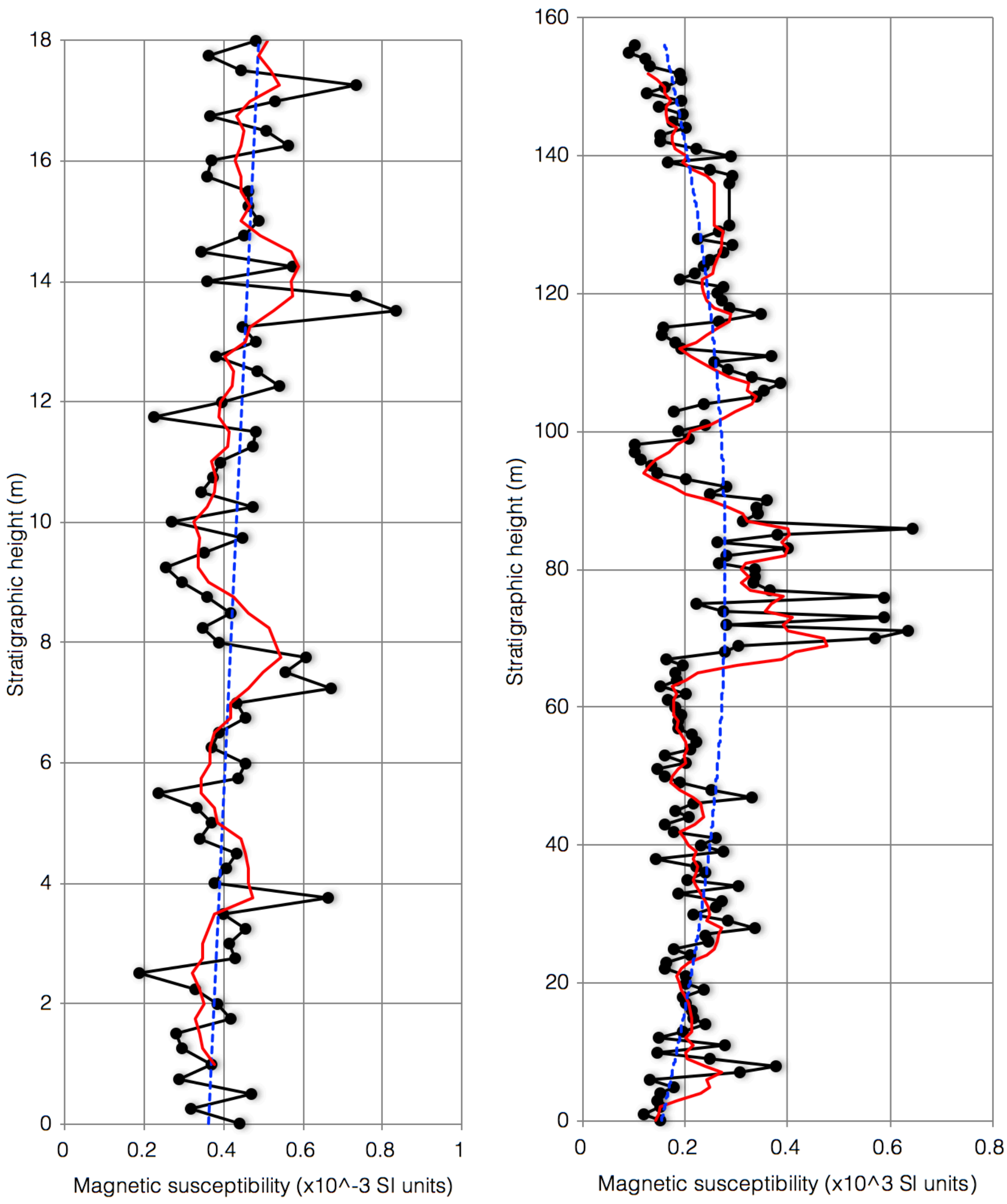

Supplementary Fig. 8. Magnetic susceptibility data and detrending in preparation for time series analysis. (Left) Magnetic susceptibility record at Holowilena Station (black) and long-term linear increase (dashed blue) used for detrending in preparation for time series analysis. A 5-point moving-average (solid red) allows for visualization of putative cyclicity even before time series analysis is conducted: note an alternation of the moving-average (solid red) back and forth across the long-term linear trend (dashed blue). (Right) Magnetic susceptibility record at Oraparinna Station (black) and long-term degree-2 polynomial trend (dashed blue) used for detrending in preparation for time series analysis. A 5-point moving-average (solid red) allows for visualization of putative cyclicity even before time series analysis is conducted: in this case, note an alternation of the moving-average (solid red) back and forth across the long-term degree-2 polynomial trend (dashed blue).

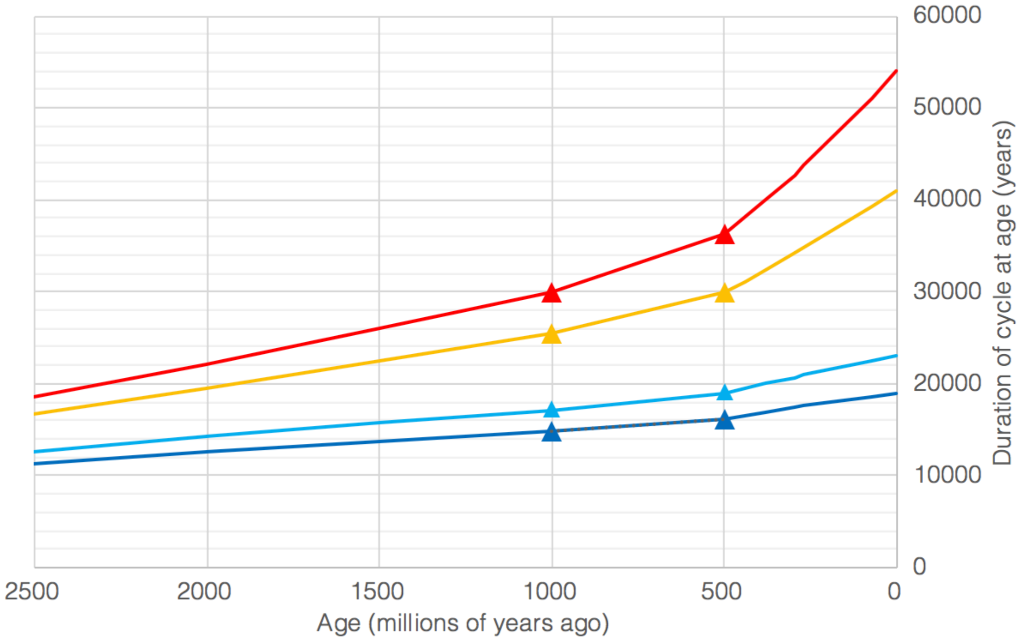

Supplementary Fig. 9. Precession and obliquity through time. Periods for precession (19 and 21 kyr) and obliquity (41 and 54 kyr) through time, as a function of decreasing spin rate and increasing Earth-Moon distance, respectively[5]. Target astronomical values used for our study (ca. 690 Ma) (Supplementary Table 3) were interpolated between the triangles. Eccentricity harmonics are assumed to be constant[6]. Obliquity bands, red and yellow; precession bands, light and dark blue.

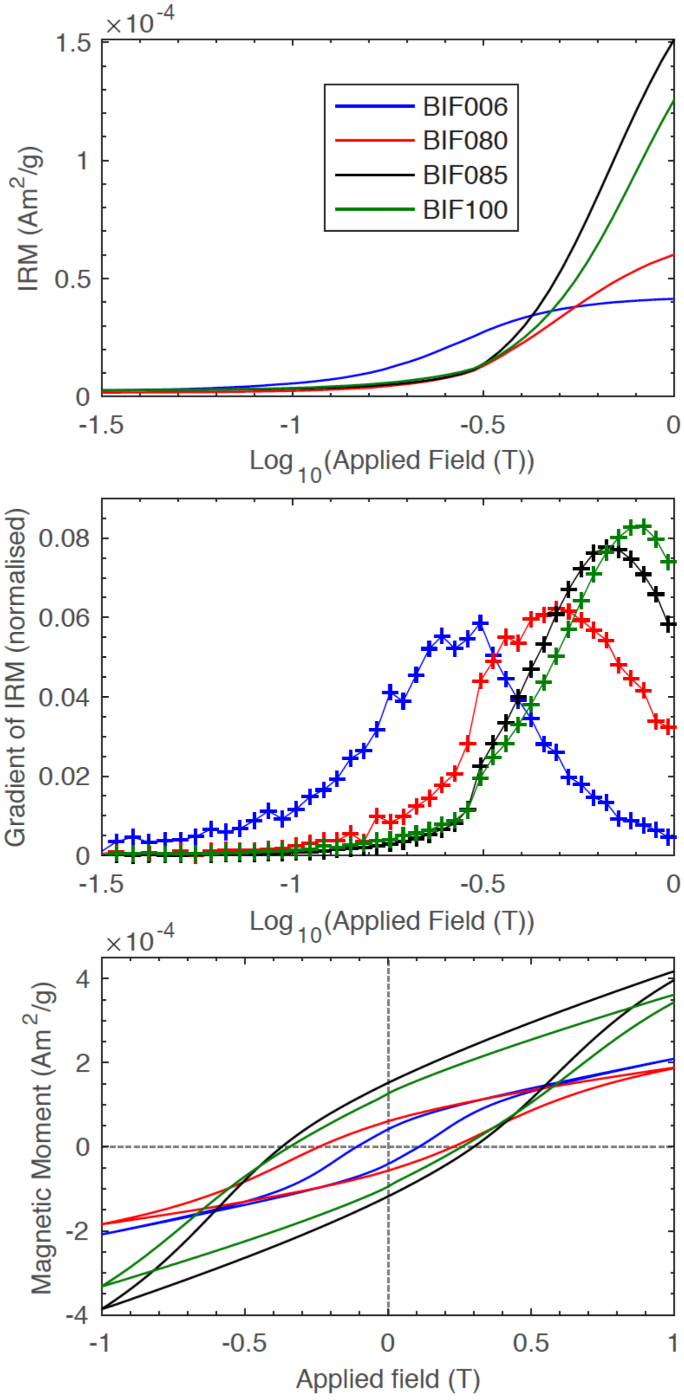

Supplementary Fig. 10. Additional rock magnetic fingerprinting. Room temperature magnetic properties of selected samples from Oraparinna (BIF080, BIF085, and BIF100) and Holowilena (BIF006) including mass normalized IRM (top), gradient of IRM (middle), and mass normalized hysteresis loops (bottom). IRM of the samples do not saturate at 1 T field, and gradient of the IRM data show dominant components with high mean coercivities of ~300 mT to ~800 mT that are indicative of hematite, consistent with “wide belt” observed on the hysteresis loops, as well as thermal susceptibility experiment results shown in Figure 5. The Holowilena BIF contains both hematite and magnetite, but the former is predominant.

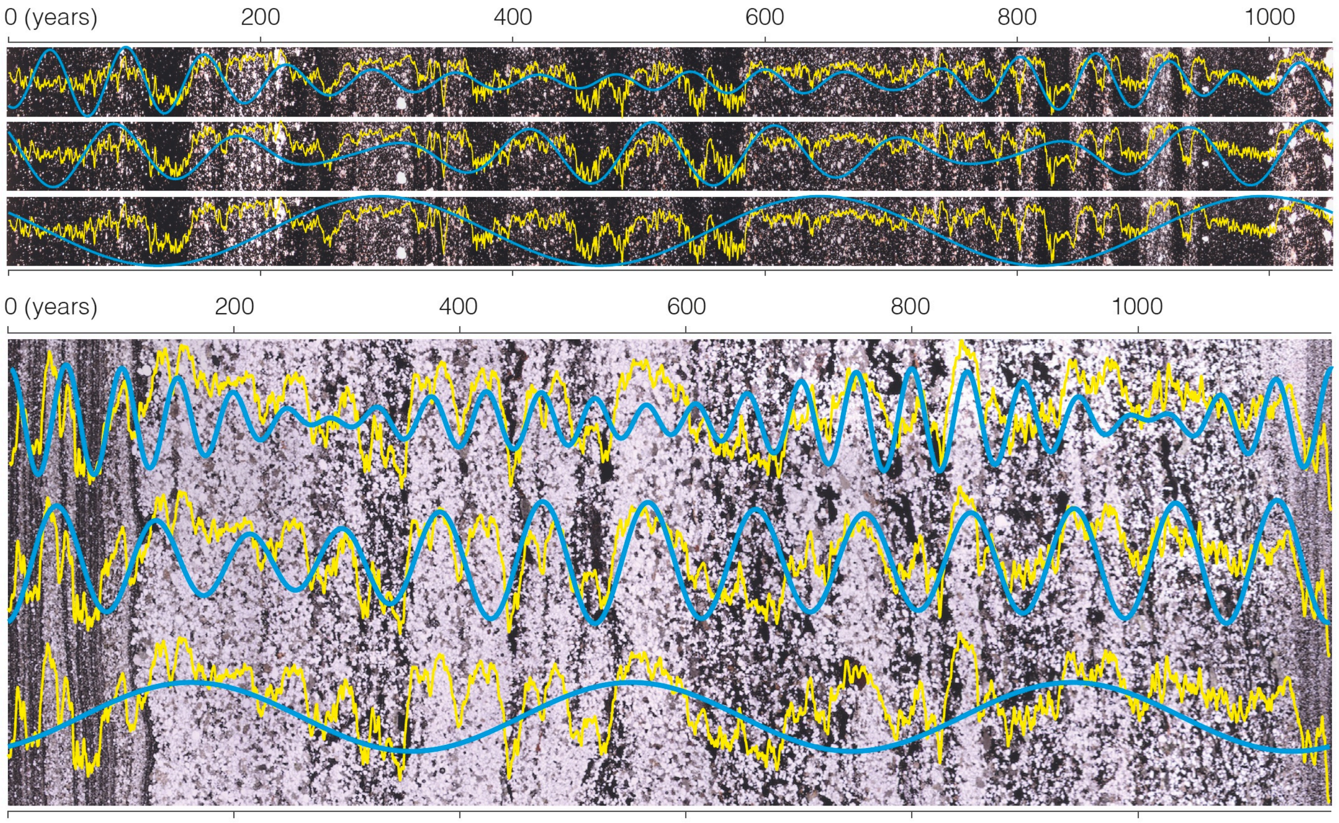

Supplementary Fig. 11. Time scale and rhythms of lithologic banding in iron formation. Raw grayscale data are yellow (light grayscale is up; dark grayscale is down). Bandpass filters are blue. Significant cycles of greatest power identified from short to long cycles (top to bottom for each sample): ~62.5, ~100, and ~450 years (top sample); ~50, ~100, and ~450 years (bottom sample). The darker sample (top; 45-mm long) is Fe-rich; the lighter sample (bottom; 49-mm long) contains more quartz grains (white in the image). The thicknesses were converted into time using the preferred sediment accumulation rate at Holowilena (Supplementary Table 3). Given the inferred sediment accumulation rates for the Holowilena BIF, the conspicuous mm-scale lithologic layering is most likely to represent suborbital glacial surges and accompanying meltwater discharge events and/or oceanic circulation cycles driven by opening and closing polynyas. Photomicrographs were taken in plane polarized light and automatically stitched together using GigaPan© Software.

Supplementary Table 1. Oraparinna locality.

Strat height (m)	Mag sus (x10^-3 SI)	Trend	Detrended
156	0.1	0.16538	-0.06538
155	0.089	0.1684	-0.0794
154	0.121	0.17138	-0.05038
153	0.131	0.17432	-0.04332
152	0.189	0.17722	0.01178
151	0.193	0.18008	0.01292
150	0.159	0.1829	-0.0239
149	0.123	0.18568	-0.06268
148	0.191	0.18842	0.00258
147	0.148	0.19112	-0.04312
146	0.196	0.19378	0.00222
145	0.175	0.1964	-0.0214
144	0.2	0.19898	0.00102
143	0.151	0.20152	-0.05052
142	0.152	0.20402	-0.05202
141	0.22	0.20648	0.01352
140	0.288	0.2089	0.0791
139	0.166	0.21128	-0.04528
138	0.247	0.21362	0.03338
137	0.293	0.21592	0.07708
136	0.285	0.21818	0.06682
130	0.286	0.2309	0.0551
129	0.265	0.23288	0.03212
128	0.225	0.23482	-0.00982
127	0.293	0.23672	0.05628
126	0.274	0.23858	0.03542
125	0.248	0.2404	0.0076
124	0.235	0.24218	-0.00718
123	0.218	0.24392	-0.02592
122	0.188	0.24562	-0.05762
121	0.273	0.24728	0.02572
120	0.261	0.2489	0.0121
119	0.272	0.25048	0.02152
118	0.286	0.25202	0.03398
117	0.347	0.25352	0.09348
116	0.264	0.25498	0.00902
115	0.157	0.2564	-0.0994

114	0.155	0.25778	-0.10278
113	0.179	0.25912	-0.08012
112	0.193	0.26042	-0.06742
111	0.367	0.26168	0.10532
110	0.256	0.2629	-0.0069
109	0.284	0.26408	0.01992
108	0.33	0.26522	0.06478
107	0.387	0.26632	0.12068
106	0.354	0.26738	0.08662
105	0.338	0.2684	0.0696
104	0.236	0.26938	-0.03338
103	0.177	0.27032	-0.09332
101	0.239	0.27208	-0.03308
100	0.186	0.2729	-0.0869
99	0.207	0.27368	-0.06668
98	0.101	0.27442	-0.17342
97	0.101	0.27512	-0.17412
96	0.112	0.27578	-0.16378
95	0.134	0.2764	-0.1424
94	0.145	0.27698	-0.13198
93	0.201	0.27752	-0.07652
92	0.28	0.27802	0.00198
91	0.247	0.27848	-0.03148
90	0.359	0.2789	0.0801
89	0.34	0.27928	0.06072
88	0.341	0.27962	0.06138
87	0.311	0.27992	0.03108
86	0.643	0.28018	0.36282
85	0.381	0.2804	0.1006
84	0.263	0.28058	-0.01758
83	0.401	0.28072	0.12028
82	0.28	0.28082	-0.00082
81	0.264	0.28088	-0.01688
80	0.335	0.2809	0.0541
79	0.337	0.28088	0.05612
78	0.334	0.28082	0.05318
77	0.366	0.28072	0.08528
76	0.588	0.28058	0.30742
75	0.221	0.2804	-0.0594
74	0.273	0.28018	-0.00718

73	0.589	0.27992	0.30908
72	0.281	0.27962	0.00138
71	0.635	0.27928	0.35572
70	0.571	0.2789	0.2921
69	0.303	0.27848	0.02452
68	0.277	0.27802	-0.00102
67	0.162	0.27752	-0.11552
66	0.194	0.27698	-0.08298
65	0.179	0.2764	-0.0974
64	0.183	0.27578	-0.09278
63	0.152	0.27512	-0.12312
62	0.2	0.27442	-0.07442
61	0.166	0.27368	-0.10768
60	0.179	0.2729	-0.0939
59	0.193	0.27208	-0.07908
58	0.186	0.27122	-0.08522
57	0.185	0.27032	-0.08532
56	0.213	0.26938	-0.05638
55	0.222	0.2684	-0.0464
54	0.21	0.26738	-0.05738
53	0.159	0.26632	-0.10732
52	0.201	0.26522	-0.06422
51	0.144	0.26408	-0.12008
50	0.16	0.2629	-0.1029
49	0.189	0.26168	-0.07268
48	0.251	0.26042	-0.00942
47	0.33	0.25912	0.07088
46	0.215	0.25778	-0.04278
45	0.18	0.2564	-0.0764
44	0.207	0.25498	-0.04798
43	0.16	0.25352	-0.09352
42	0.176	0.25202	-0.07602
41	0.258	0.25048	0.00752
40	0.23	0.2489	-0.0189
39	0.274	0.24728	0.02672
38	0.141	0.24562	-0.10462
37	0.221	0.24392	-0.02292
36	0.238	0.24218	-0.00418
35	0.203	0.2404	-0.0374
34	0.303	0.23858	0.06442

33	0.185	0.23672	-0.05172
32	0.27	0.23482	0.03518
31	0.258	0.23288	0.02512
30	0.215	0.2309	-0.0159
29	0.283	0.22888	0.05412
28	0.335	0.22682	0.10818
27	0.239	0.22472	0.01428
26	0.245	0.22258	0.02242
25	0.178	0.2204	-0.0424
24	0.21	0.21818	-0.00818
23	0.163	0.21592	-0.05292
22	0.161	0.21362	-0.05262
21	0.202	0.21128	-0.00928
20	0.202	0.2089	-0.0069
19	0.236	0.20648	0.02952
18	0.196	0.20402	-0.00802
17	0.202	0.20152	0.00048
16	0.211	0.19898	0.01202
15	0.216	0.1964	0.0196
14	0.24	0.19378	0.04622
13	0.194	0.19112	0.00288
12	0.147	0.18842	-0.04142
11	0.278	0.18568	0.09232
10	0.145	0.1829	-0.0379
9	0.247	0.18008	0.06692
8	0.377	0.17722	0.19978
7	0.306	0.17432	0.13168
6	0.131	0.17138	-0.04038
5	0.178	0.1684	0.0096
4	0.15	0.16538	-0.01538
3	0.145	0.16232	-0.01732
2	0.15	0.15922	-0.00922
1	0.118	0.15608	-0.03808
0	0.15	0.1529	-0.0029

.

Supplementary Table 2. Holowilena locality.

Strat height (m)	Mag sus (x10^-3 SI)	Trend	Detrended
0	0.441	0.3593	0.0817
0.25	0.315	0.3611	-0.0461
0.5	0.469	0.3629	0.1061
0.75	0.286	0.3647	-0.0787
1	0.369	0.3665	0.0025
1.25	0.293	0.3683	-0.0753
1.5	0.281	0.3701	-0.0891
1.75	0.416	0.3719	0.0441
2	0.385	0.3737	0.0113
2.25	0.326	0.3755	-0.0495
2.5	0.185	0.3773	-0.1923
2.75	0.428	0.3791	0.0489
3	0.413	0.3809	0.0321
3.25	0.455	0.3827	0.0723
3.5	0.4	0.3845	0.0155
3.75	0.664	0.3863	0.2777
4	0.376	0.3881	-0.0121
4.25	0.407	0.3899	0.0171
4.5	0.433	0.3917	0.0413
4.75	0.338	0.3935	-0.0555
5	0.367	0.3953	-0.0283
5.25	0.333	0.3971	-0.0641
5.5	0.234	0.3989	-0.1649
5.75	0.434	0.4007	0.0333
6	0.454	0.4025	0.0515
6.25	0.367	0.4043	-0.0373
6.5	0.386	0.4061	-0.0201
6.75	0.453	0.4079	0.0451
7	0.431	0.4097	0.0213
7.25	0.67	0.4115	0.2585
7.5	0.554	0.4133	0.1407
7.75	0.608	0.4151	0.1929
8	0.388	0.4169	-0.0289
8.25	0.346	0.4187	-0.0727
8.5	0.416	0.4205	-0.0045
8.75	0.356	0.4223	-0.0663
9	0.295	0.4241	-0.1291

9.25	0.255	0.4259	-0.1709
9.5	0.349	0.4277	-0.0787
9.75	0.448	0.4295	0.0185
10	0.269	0.4313	-0.1623
10.25	0.473	0.4331	0.0399
10.5	0.344	0.4349	-0.0909
10.75	0.373	0.4367	-0.0637
11	0.39	0.4385	-0.0485
11.25	0.472	0.4403	0.0317
11.5	0.479	0.4421	0.0369
11.75	0.224	0.4439	-0.2199
12	0.396	0.4457	-0.0497
12.25	0.538	0.4475	0.0905
12.5	0.483	0.4493	0.0337
12.75	0.378	0.4511	-0.0731
13	0.48	0.4529	0.0271
13.25	0.447	0.4547	-0.0077
13.5	0.835	0.4565	0.3785
13.75	0.734	0.4583	0.2757
14	0.357	0.4601	-0.1031
14.25	0.575	0.4619	0.1131
14.5	0.344	0.4637	-0.1197
14.75	0.45	0.4655	-0.0155
15	0.489	0.4673	0.0217
15.25	0.463	0.4691	-0.0061
15.5	0.46	0.4709	-0.0109
15.75	0.357	0.4727	-0.1157
16	0.367	0.4745	-0.1075
16.25	0.563	0.4763	0.0867
16.5	0.507	0.4781	0.0289
16.75	0.366	0.4799	-0.1139
17	0.528	0.4817	0.0463
17.25	0.735	0.4835	0.2515
17.5	0.444	0.4853	-0.0413
17.75	0.36	0.4871	-0.1271
18	0.479	0.4889	-0.0099

Supplementary Table 3. Comparison of observed and orbital cycles

Cycle (m)	(kyr)	Target cycle	(kyr)	Misfit (kyr)
0.95*	23.1	precession	17.007**	6.1
4.00*	97.1	95-kyr eccentricity	95	2.1
4.37*	106.1	short eccentricity	112.25***	-6.2
17.5	405.1	long eccentricity	405.1	0.0
32.5	752.3	-		2.00
52.9	1224.5	~1.2 Myr obliquity		
108	2500.0	~2.4 Myr eccentricity		
140	3240.7	-		

All cycles from Oraparinna (sediment accumulation rate = 4.32 cm/kyr),

expect those from *Holowilena (sediment accumulation rate = 4.12 cm/kyr).

**Mean of two precessional components (15.689 and 18.325 kyr; fig. S9).

***Mean of short eccentricity components (95, 99, 124, and 131 kyr)

Supplementary References

1. Cox, G. M. *et al.* A model for Cryogenian iron formation. *Earth and Planatary Science Letters* **433**, 280-292 (2016).
2. Lechte, M. A. *et al.* Subglacial meltwater supported aerobic marine habitats during Snowball Earth. *Proceedings of the National Academy of Sciences* **116**, 25478-25483 (2019).
3. Preiss, W. V. The Adelaide Geosyncline--late Proterozoic stratigraphy, sedimentation, palaeontology and tectonics. *Geological Survey South Australia Bulletin* **53**, 438 (1987).
4. Le Heron, D. P., Cox, G. M., Trundley, A. & Collins, A. S. Sea ice-free conditions during the Sturtian glaciation (early Cryogenian), South Australia. *Geology* **39**, 31-34 (2011).
5. Berger, A. & Loutre, M. F. Astronomical forcing through geological time. *Special Publications of the International Association of Sedimentology* **19**, 15-24 (1994).
6. Hinnov, L. A. Cyclostratigraphy and its revolutionizing applications in the earth and planetary sciences. *Geological Society of America Bulletin* **125**, 1703-1734 (2013).
